# Chronic sustained hypoxia alters the pattern of diaphragm electrical activity in anaesthetized rats

**DOI:** 10.1113/EP092211

**Published:** 2025-02-24

**Authors:** Jamal Khalilpour, Mohammad Reza Alipour, Parviz Shahabi

**Affiliations:** ^1^ Drug Applied Research Center Tabriz University of Medical Sciences Tabriz Iran; ^2^ National Medical Emergency Organization Ministry of Health & Medical Education Tehran Iran; ^3^ Stem Cell Research Center Tabriz University of Medical Sciences Tabriz Iran

**Keywords:** chronic sustained hypoxia, diaphragm electromyography, diaphragm muscle, power spectral density, respiratory plasticity

## Abstract

Chronic sustained hypoxia (CSH) is known to induce functional and structural changes in the respiratory system. The diaphragm, as the main inspiratory muscle of mammals, is particularly important in the neuromotor regulation of respiration. Diaphragm electromyography (dEMG) records the sum of motor unit action potentials (MUAP) and provides information regarding motor unit recruitment and frequency coding during muscle contraction. We aimed to assess changes in dEMG activity following CSH. Herein, eight male Wistar rats (2–3 months) were subjected to CSH (10 ± 0.5% O_2_) for 10 successive days. *In vivo* dEMG recording was employed to assess changes in the diaphragm electrical activity. Filtered and rectified dEMGs were used for further analyses. Findings showed that CSH for 10 consecutive days significantly changed the pattern of dEMG signals. The slope of the rising phase of RMS‐enveloped dEMG bursts was much steeper in CSH rats compared to normoxic control rats (rise time: 373 vs. 286 ms; *P *= 0.005). Burst frequency significantly decreased following CSH (59 vs. 42 bursts/min; *P* = 0.0001), which was associated with a significant increase in burst amplitude (*P* = 0.039) and inter‐burst duration (0.65 vs. 0.88 s; *P* = 0.041). Power spectral density analyses showed that the mean frequency (293 vs. 266 Hz; *P* = 0.033) and high‐frequency to low‐frequency power ratio (*P* = 0.009) of dEMG signals significantly declined in CSH rats. Notably, the regularity of frequency and amplitude of dEMG signals did not change significantly following CSH.

## Abstract (highlights)

- **What is the central question of this study?**
  What are the effects of chronic sustained hypoxia on the pattern and characteristics of the electrical activity of the diaphragm muscle?
- **What is the main finding and its importance?**
  The treatment of rats with chronic sustained hypoxia (CSH: 10% O_2_ for 10 consecutive days) altered the pattern and characteristics of the diaphragm electromyography (dEMG). The shape of dEMG burst activity changed from a ramp‐like augmenting pattern to a bell‐shaped pattern following CSH. Moreover, CSH changed frequency, duration, amplitude and frequency content of dEMG bursts.

## 1 INTRODUCTION

The respiratory system of mammals responds to hypoxic conditions by employing several physiological mechanisms to maintain normal arterial O_2_ levels. The hypoxic ventilatory response (HVR) of the respiratory system is related to the duration (acute or chronic), intensity (mild to intense) and pattern (sustained or intermittent) of hypoxic exposure. It involves various mechanisms that can facilitate or suppress ventilatory components (tidal volume and respiratory frequency) over the course of seconds to years (reviewed in detail by Pamenter & Powell, 2016; Powell et al., 1998). In response to chronic sustained hypoxia (CSH; days to weeks), a unique type of HVR termed ventilatory acclimatization to hypoxia (VAH) occurs, which is a time‐dependent increase in ventilation (Pamenter & Powell, 2016; Powell et al., 1998). To achieve this, the respiratory system undergoes functional and structural changes to ensure adequate ventilation during sustained hypoxia. Any component of the respiratory system, from rhythm generating respiratory neurons in the medulla to respiratory muscles, may contribute to VAH (Pamenter & Powell, 2016). Respiratory plasticity following CSH has been reported in respiratory‐related neural circuits (Arbogast et al., 2016; Dwinell & Powell, 1999; Faul et al., 2020; Forster et al., 1976, 1974; Morinaga et al., 2016; Powell et al., 2000), as well as in respiratory muscles, including the diaphragm (Degens et al., 2010; Gamboa & Andrade, 2012; McMorrow et al., 2011; Mortola & Naso, 1995; Navarrete‐Opazo & Mitchell, 2014; Shiota et al., 2004).

The diaphragm, as the main inspiratory pump muscle of mammals, is particularly important in the neuromotor regulation of respiration (Sieck, 1991). Similar to other striated muscles, the diaphragm exhibits a remarkable capacity for plasticity in both health and disease (Greising et al., 2018). In sustained hypoxic conditions, the diaphragm needs to adapt its contractile elements to the increased inspiratory load imposed by the respiratory network to maintain sustaining contractions. Indeed, structural and functional changes in the diaphragm muscle have been reported following CSH (Degens et al., 2010; El‐Khoury et al., 2003; Gamboa & Andrade, 2010, 2012; Lewis & O'Halloran, 2016; McMorrow et al., 2011; Mortola & Naso, 1995; Shiota et al., 2004). There are some reports of sustained hypoxia‐induced maladaptive changes, such as decreased muscle fibre cross‐sectional areas and muscle force, in diaphragm muscle (Degens et al., 2010; Gamboa & Andrade, 2012; Lewis et al., 2016; McMorrow et al., 2011; Shiota et al., 2004). Conversely, there are also adaptive changes in diaphragm muscle following sustained hypoxia. For instance, the diaphragm fatigue tolerance is preserved or increased in response to sustained hypoxia lasting 4–6 weeks in rats and mice (Gamboa & Andrade, 2009, 2012; Lewis et al., 2015; McMorrow et al., 2011). Furthermore, it was found that exposure to CSH treatment also increases the tolerance of rat diaphragm muscle in response to acute severe hypoxia, suggesting enhanced force‐generating capacity during severe hypoxia following CSH (Lewis et al., 2015).

Diaphragm electromyography (dEMG) records the sum of motor unit action potentials (MUAP) and represents the central respiratory drive (Lindstrom & Magnusson, 1977). It provides details on the frequency coding and recruitment of motor units during muscle contraction (Keenan et al., 2005; Lowery, 2009; Seven et al., 2013). The amplitude of dEMG is not indicative of the diaphragm contractility or the real amount of force generated by the muscle; however, the root mean square (RMS) of dEMG is highly correlated with the generated transdiaphragmatic pressure during muscle contraction (Mantilla et al., 2010). There are many reports in the literature regarding changes in dEMG in response to acute or short‐term sustained hypoxia (Corne et al., 2000; Easton et al., 1995; Moss & Laferrière, 1998; Navarrete‐Opazo & Mitchell, 2014; Pierce & Clancy, 2001; Xie et al., 1993). However, there are insufficient studies on dEMG alteration following CSH. Therefore, we sought to assess changes in the pattern and characteristics of dEMG signals following chronic sustained hypoxia in anaesthetized rats.

## 2 METHODS

### 2.1 Ethical approval

This study followed the ethical requirements for animal work set by *Experimental Physiology* (Grundy, 2015). All experiments were performed in agreement with the guidelines of the Ethics Committee of Tabriz University of Medical Sciences, Iran, for the care and use of laboratory animals (IR.TBZMED.VCR.REC.1399.404).

### 2.2 Animals and study design

Animals were purchased from the Pasteur Institute, Tehran, Iran. In this study, 16 male Wistar rats (weight: 200–300 g; age: 2–3 months) were randomly divided into two groups: (1) a control group (Ctrl), and (2) a chronic sustained hypoxic group (CSH). The rats were brought to the experiment room 2 days before the induction of chronic sustained hypoxia to become accustomed to the new environment. The animals were housed in standard cages in a room with controlled temperature (22–24°C), humidity (40–60%), and light period (12 h dark started at 20.00 h and 12 h light started at 08.00 h). All the animals had free access to food and water ad libitum. On the third day, the induction of chronic hypoxia (10 consecutive days) started for CSH group. Three days after exposure to CSH (days 14–15), direct electrophysiological recordings from the diaphragm muscle (dEMG) were carried out.

### 2.3 CSH induction protocol

For this, we employed a previously described CSH induction protocol by our institute (Khalilpour et al., 2024). Briefly, hypoxic animals were placed in a Plexiglas hypoxic chamber for 10 consecutive days (24 h per day). The hypoxic chamber (100 cm width, 200 cm height, and 100 cm depth) had the capacity to induce hypoxia for eight adult rats, simultaneously. The rats had access to water and food ad libitum during the period of hypoxia induction. Low oxygen air (10 ± 0.5% O_2_) was made with the GO_2_Altitude system (Bio MedTech Australia Pty., Ltd, Moorabbin, Victoria, Australia) and directed to the chamber through an air tube. The chamber was equipped with an air‐circulating system, and the carbon dioxide produced by the metabolism escaped through an opening that was embedded in the lower part of the chamber wall. An oxygen sensor (OOM202, Envitec, Germany) continuously detected the oxygen level in the chamber to keep it constant at around 10%. The system reduced the oxygen level in the chamber from 21% to 10% within around 30 min. The chamber door was opened for only 20 min daily to add food and water and clean the cages. Control rats were also kept in a similar Plexiglas chamber for 10 consecutive days (24 h per day), breathing 21% O₂ in the same time frame.

### 2.4 In vivo direct electrophysiological recordings from diaphragm

All dEMG recordings were obtained in normoxic conditions. For this purpose, the animals were anaesthetized with an intraperitoneal injection of urethane (1.5 g/kg). Then, the spontaneously breathing animals were placed in the recording cage in a supine position. The rectal temperature was continuously monitored and maintained between 36°C and 37.5°C by placing the rats on a heating pad. Then, a lateral incision was made in the left side of the abdomen to expose the lower left side of the diaphragm. For electrical activity recording from the diaphragm muscle, a pair of parallel stainless‐steel Teflon‐coated wires was used; 5 mm from their distal ends, they were stripped, and an interelectrode distance (ID) of 3 mm was created between the stripped ends. The wires were placed into the left costal part of the diaphragm and fixed to the adjacent skin with a knot using a 4/0 silk thread. For each subject 30 min dEMG activity was acquired employing a multifunctional data acquisition system (NI USB‐6221, National Instruments, Austin, TX, USA). Recordings were amplified (with a gain of 1000) and sampled at 20 kHz. At the end of electrophysiological recordings (30 min after electrophysiological recordings), the animals were euthanized with lethal dose of urethane (2 g/kg).

### 2.5 Analysis of dEMG signals

**FIGURE 1:**
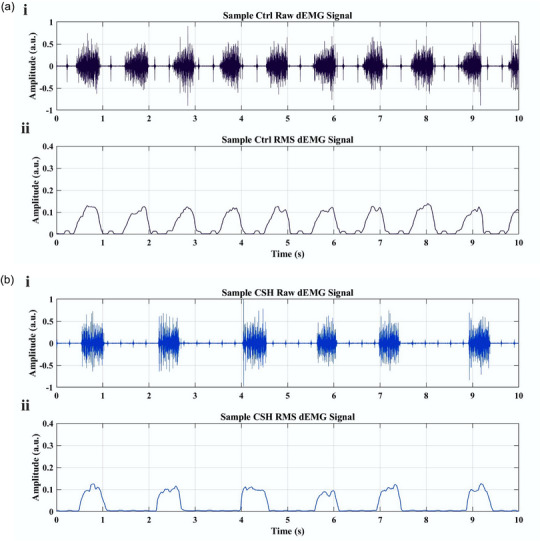
Sample filtered raw dEMG signals (i) and their corresponding RMS‐enveloped rectified signals (ii) recorded from control normoxic (a) and CSH rats (b).

**FIGURE 2:**
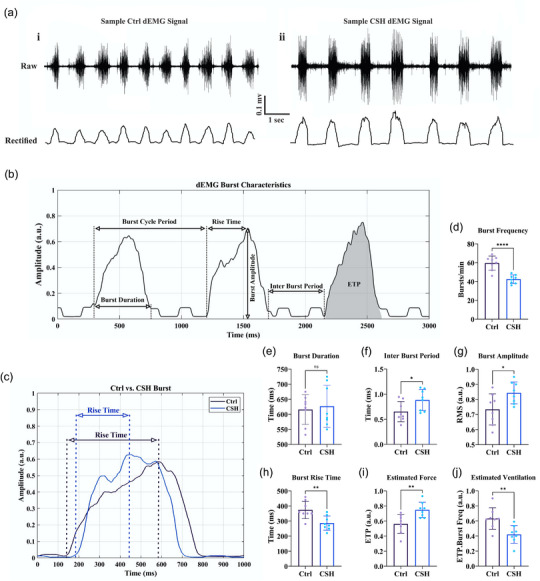
Changes in the pattern and characteristics of dEMG signals following CSH. (a) Sample raw dEMG (upper panel) and their corresponding rectified (lower panel) traces recorded from normoxic (i) and hypoxic (ii) rats. (b) Measured parameters of dEMG signals from RMS‐enveloped dEMG signals. (c) Comparison of the shape and rise time of dEMG bursts between control (Ctrl) and CSH rats. Note the shorter rise time in CSH dEMG burst. (d–j) Changes in dEMG characteristics (burst frequency, burst duration, inter‐burst period, burst amplitude, burst rise time, estimated force and estimated minute ventilation) subtracted from RMS‐enveloped dEMG signals following CSH. ETP, EMG‐time product. Values are presented as means ± SD. An independent *t*‐test was employed to compare the mean of variables between groups (*n* = 8 in each group; ns, not significant; *P < 0.05, **P < 0.01, ***P < 0.001, ****P < 0.0001, compared to Ctrl group).

**FIGURE 3:**
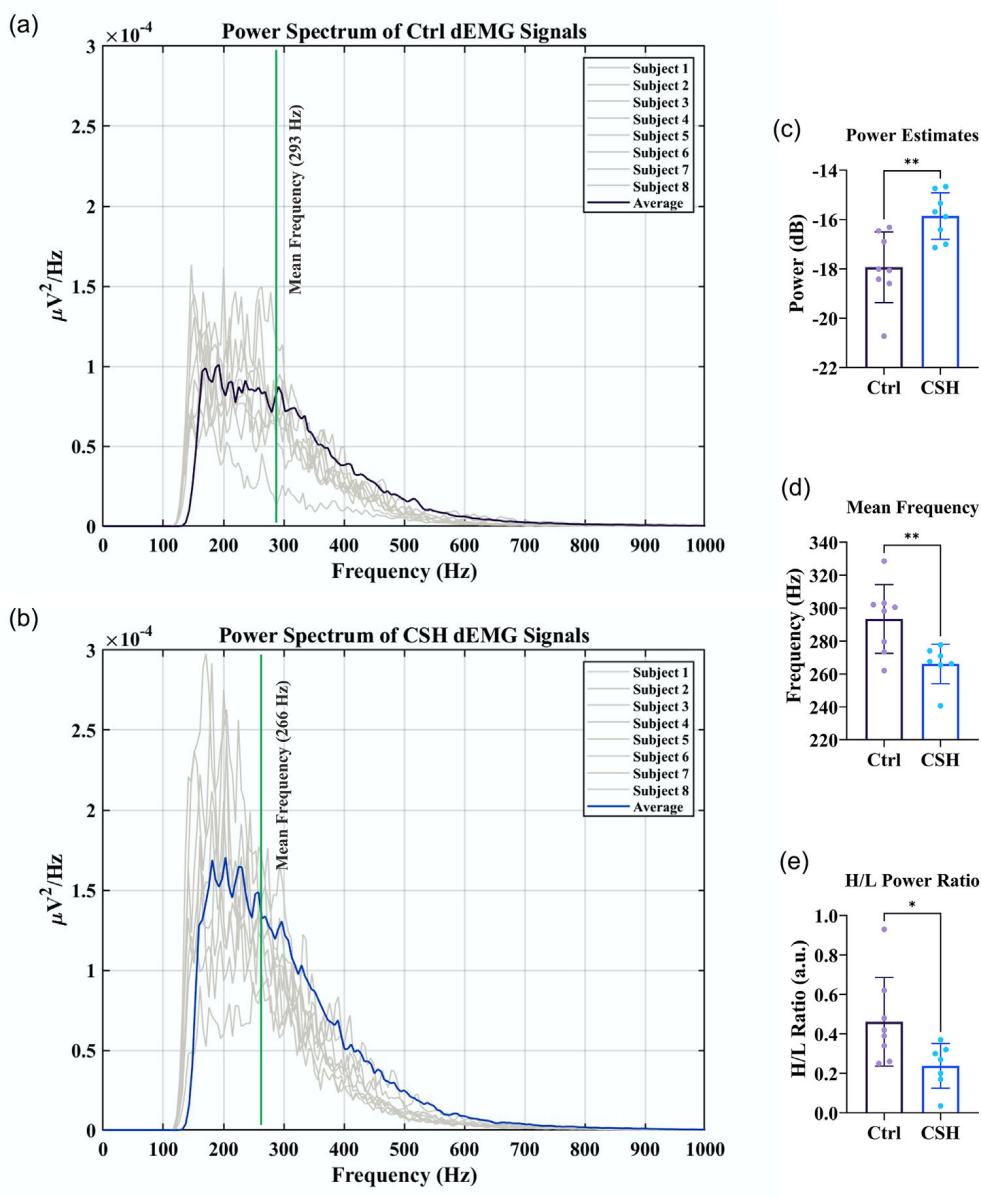
Power spectral density (PSD) analyses of dEMG signals. (a, b) PSD was estimated by employing Welch's method for control (a) and CSH (b) dEMG signals. (c–e) Bar graphs showing changes in the total power (c), the mean frequency (d), and the high‐frequency power/low‐frequency power ratio (e) following CSH. Values are presented as means ± SD. An independent *t*‐test was employed to compare the mean of variables between groups (*n* = 8 in each group; ns, not significant; *P < 0.05, **P < 0.01, compared to Ctrl group.

After data acquisition, all preprocessing steps for raw signals were done offline using Signal Processing Toolbox 9.2 (The MathWorks, Natick, MA, USA). Raw dEMG signals were initially bandpass filtered (100 to 1000 Hz) and a 5‐min epoch was selected for each subject. To remove ECG artifacts from dEMG signals, a wavelet‐based adaptive filter was applied (Zhan et al., 2010). This method utilizes discrete wavelet transform to decompose dEMG signals into approximation (low pass components) and detail coefficients (high pass components). We used a 4th order Daubechies (db4) wavelet to decompose dEMG signals to level 5. Then, dEMG signals were filtered in the wavelet space by employing an adaptive threshold. Finally, the wavelet‐filtered dEMG signals were reconstructed using the inverse discrete wavelet transform. Filtered and cleaned dEMGs were normalized (against the maximum amplitude of sighing inspiratory bursts during recording), rectified and smoothed for inspiratory burst detection and estimating dEMG characteristics (e.g., burst frequency, burst duration, burst rise time, burst amplitude, etc.) (Figure 2). Figure 1 shows sample raw dEMG signals and their corresponding RMS‐enveloped rectified signals recorded from control normoxic (Figure 1a) and CSH rats (Figure 1b). To estimate the intensity of muscle activation, the area under the inspiratory waveform (i.e., dEMG‐time product; Graßhoff et al., 2021, 2023; Figure 3a) of 30 successive dEMG bursts for each subject was calculated. Furthermore, using a custom MATLAB (The MathWorks) code, the total power of dEMG signals and the power content of high‐frequency (the power within the highest (4th) quartile band of frequencies in the eupneic PSD: 675–1000 Hz) and low‐frequency (the power within the lowest (1st) quarter of eupneic PSD: 100–225 Hz) of dEMGs were estimated (Seven et al., 2013). Then, a high‐frequency to low‐frequency power ratio (H/L ratio) was calculated for each subject. In addition, the mean frequency of the dEMG signal was estimated using the ‘meanfreq’ function in MATLAB. Moreover, for a graphical review of dEMG signals in frequency domain, we employed the Matlab ‘pwelch’ power spectrum estimating function.

### 2.6 Statistical analysis

Statistical analyses were performed using GraphPad Prism version 9 (GraphPad Software, Boston, MA, USA). An independent Student's *t*‐test was employed to compare the mean burst duration, burst frequency, burst rise time, burst amplitude, inter‐burst duration and intensity of muscle activation, as well as the total power, mean frequency and H/L ratio of dEMG signals, between two groups. *P *< 0.05 was considered statistically significant. All values were presented as means ± SD.

## 3 RESULTS

### 3.1 CSH changed the pattern of dEMG bursts

Three days after CSH treatment, in vivo diaphragm electromyography (dEMG) recordings were obtained from anaesthetized spontaneously breathing rats during normoxic (room air) conditions. The dEMG bursts of control rats have a ramp‐like augmenting pattern with a slight slope in the rising phase. We did not observe the same pattern in CSH dEMG bursts, where the slope of the rising phase was much steeper compared to dEMG bursts of normoxic rats (Figure 2c). This finding was confirmed by calculating the rising time of dEMG bursts (burst rise time), which was significantly lower in CSH bursts (Figure 2h; 373 vs. 286 ms; *P *= 0.005; eight animals in each group).

### 3.2 CSH changed the characteristics of dEMG bursts

Chronic exposure to hypoxia for 10 days resulted in a significant decrease in normoxic burst frequency (Figure 2d; 59 ± 7.5 vs. 42 ± 4.5 bursts/min; *P* = 0.0001; *n* = 8 in each group), which was primarily due to a significant decrease in inter‐burst duration (Figure 2f; time: 0.65 ± 0.2 vs. 0.88 ± 0.2 s; *P *= 0.0412; *n* = 8 in each group). Notably, the change in burst duration following CSH treatment was not statistically significant (Figure 2e; time: 615 ± 49 vs. 626 ± 69 ms; *P* = 0.0727). Furthermore, CSH led to the increased amplitude of dEMG bursts (Figure 2h; amplitude: 0.59 ± 0.09 vs. 0.68 ± 0.06 a.u.; *P* = 0.0393; *n* = 8 in each group) and the area under the dEMG burst curve (Figure 2i; ETP: 0.60 ± 0.08 vs. 0.70 ± 0.06 a.u.; *P* = 0.0275; *n* = 8 in each group), suggesting enhanced force generation by the diaphragm muscle (Graßhoff et al., 2023). Moreover, when we measured the area under the dEMG bust curve during 1 min of dEMG signals (ETP × burst frequency) to estimate minute ventilation, it was found that the estimated ventilation was significantly lower in CSH rats (Figure 2j; ETP **×** Burst Freq: 0.63 ± 0.14 vs. 0.42 ± 0.11 a.u.; *P* = 0.0063; *n* = 8 in each group).

### 3.3 CSH changed the frequency content of dEMG signals

It has been shown that hypoxia alters the power spectral density (PSD), mean frequency (MNF) and ratio of high‐frequency power to low‐frequency power (H/L ratio) of the diaphragm EMG (Aldrich et al., 1983; Seven et al., 2013; Watchko et al., 1987). Therefore, we analysed dEMG signals in the frequency domain to estimate the power (total power and the power of 1st and 4th quarter of frequency content) and power spectral density of dEMG signals. For this, 20 successive eupnoeic inspiratory efforts (dEMG bursts) were analysed for each subject. Since the diaphragm contractions are dynamic and consequently dEMG signals are non‐stationary, each dEMG burst was divided into shorter 0.05 s time epochs. Then, by employing Welch's method (‘pwelch’ function in MATLAB; hamming window, 1000 samples width and 50% overlapping) the power spectral density of each epoch was estimated and the average power spectrum was estimated for each subject (McManus et al., 2020). Moreover, we used ‘meanfreq’ and ‘bandpower’ functions in MATLAB to estimate changes in MNF and H/L ratio, respectively. We found that CSH for 10 consecutive days significantly increased the total power of dEMG signals (Figure 3c; dEMG power: ‒17.94 ± 1.4 vs. ‒15.86 ± 0.94 dB; *P* = 0.0041; *n* = 8 in each group). This finding was verified by PSD graphical analysis of dEMG signals (Figure 3a, b). Furthermore, CSH rats showed a significant decrease in the mean frequency of dEMG signals (Figure 3a, b, d; mean frequency: 293 ± 20 vs. 266 ± 12 Hz; *P* = 0.0095; *n* = 8 in each group). Moreover, the ratio of high‐frequency power to low‐frequency power declined significantly in CSH animals compared to normoxic controls (Figure 3d; H/L ratio: 0.46 ± 0.22 vs. 0.23 ± 0.11 a.u.; *P* = 0.0334; *n* = 8 in each group).

### 3.4 CSH increased the irregularity of dEMG signals

**FIGURE 4:**
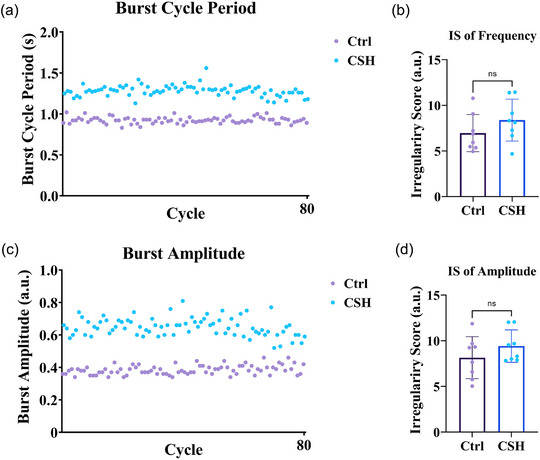
Changes in the regularity of dEMG signals following CSH. (a) Comparison of burst period regularity between Ctrl and CSH dEMG signals calculated for 80 successive bursts (cycles). (b) Quantitative analyses of the irregularity score for frequency (IS_Freq_) between Ctrl and CSH groups. (c) Comparison of burst amplitude regularity between Ctrl and CSH dEMG signals calculated for 80 successive bursts (cycles). (d) Quantitative analyses of the irregularity score for burst amplitude (IS_Amp_) between Ctrl and CSH groups. Note that CSH for 10 days increased the irregularity of frequency and amplitude; however, these changes were not statistically significant. Values are presented as means ± SD. An independent *t*‐test was employed to compare the average of variables between groups (*n* = 8 in each group; ns, not significant compared to Ctrl group).

It has been reported that respiratory rhythmogenesis becomes irregular following hypoxic exposure (Garcia et al., 2016; Zanella et al., 2014). Since dEMG represents the output of the central respiratory drive, we aimed to estimate the regularity of dEMG signals following CSH (Figure 4).

To do so, we calculated the irregularity score of frequency (IS_Freq_) using a mathematical formula previously described by Garcia et al. (2016) and Telgkamp et al. (2002). IS_Freq__ *_N_* = 100 × ABS (*P_N_*–*P_N_*_−1_)/*P_N_*_−1_; where IS_Freq._ *_N_* is the irregularity score of the dEMG burst *N*, ABS is the absolute value, *P_N_* is the burst cycle period of burst *N*, and *P_N_*_−1_ is the burst cycle period of the proceeding dEMG burst (Figure 2b). The IS_Freq_ was calculated for 80 successive bursts for each subject (Figure 4). The greater the IS_Freq_, the more irregular the dEMG burst activity. The same formula was also used for estimating the irregularity score of dEMG burst amplitude (IS_Amp_). In this case, *P* (burst cycle period) in the formula was replaced with *A* (amplitude of bursts). We found that CSH led to increased IS_Freq_ and IS_Amp_; however, these changes were not statistically significant (Figure 4a, b; IS_Freq_: 6.94 ± 2.0 vs. 8.37 ± 2.2 a.u.; *P* = 0.2083; IS_Amp_: 8.14 ± 2.2 vs. 9.41 ± 1.7 a.u.; *P* = 0.2363; *n* = 8 in each group).

## 4 DISCUSSION

In the present study, we report changes in the pattern (shape), characteristics (e.g., burst frequency, inter‐burst duration, burst rise time, burst RMS amplitude, etc.), and frequency contents of dEMG signals following 10 days of chronic exposure to hypoxia.

We found that the pattern of dEMG bursts has changed following CSH. The electrical activity of the diaphragm reflects the neural respiratory drive arising from brainstem respiratory neurons traveling through the phrenic nerve and reaching the diaphragm muscle. Therefore, the altered pattern of dEMG bursts following CSH, at least in part, can be attributable to changes in the pattern of the inspiratory signal arising from rhythm‐generating neurons within the pre‐Bötzinger complex (preBötC). Indeed, changes in the pattern of inspiratory rhythm bursts following hypoxic exposure have been reported by numerous studies (Lieske et al., 2000; Peña, 2008; Solomon, 2004; Viemari et al., 2011). For instance, in response to severe hypoxia, the inspiratory rhythm pattern transitions from a bell‐shaped pattern to a fast‐rising steeper form, which is a characteristic of gasping (Lieske et al., 2000; Peña, 2008). It is proposed that during acute hypoxia, the reconfiguration of the preBötC neuronal circuit results in gasping rhythmogenesis (Juárez‐Vidales et al., 2021; Lieske et al., 2000; Nieto‐Posadas et al., 2014; Potts & Paton, 2006). Whether the pattern of the respiratory signal also changes during or following CSH remains to be determined and requires direct electrophysiological recordings from preBötC neural circuits or the phrenic nerve. Moreover, the pattern of an EMG signal is also determined by the recruitment pattern of motor units and the shape of motor unit action potentials (MUAP) recorded from active motor units (Luca, 1979; McManus et al., 2020; Moritani et al., 2004). In turn, the shape of MUAPs depends on the motor unit properties, such as the number of muscle fibres innervated by the motoneuron and fibre type (Rodriguez‐Falces, 2015). For instance, motor units with larger muscle fibres conduct action potential with a higher velocity and give rise to a shorter MUAP with a steeper rise phase (McManus et al., 2020). Thus, the changed pattern of the dEMG signal following CSH may reflect alterations in the recruitment pattern of diaphragm motor units by respiratory motoneurons.

As stated before, during prolonged sustained hypoxia, the respiratory system of mammals responds with a time‐dependent increase in ventilation (VAH) (Pamenter & Powell, 2016; Powell et al., 1998). After CSH, the ventilation remains elevated for hours to weeks, depending on the animal species, which is called ventilatory deacclimatization from hypoxia (VDH) (Bisgard & Neubauer, 1995; Pamenter & Powell, 2016; Powell et al., 1998). We found a significant decrease in estimated minute ventilation from dEMG signals, recorded 3 days after CSH cessation, that is against VDH reported in human and animal models (Dempsey et al., 1979; Fatemian & Robbins, 1998; Moya & Powell, 2018; Sato et al., 1994; Tansley et al., 1998). However, in rats and humans, VAH and VDH are mainly due to increased tidal volume (Aaron & Powell, 1993; Forster et al., 1974), which is consistent with increased RMS burst amplitude and area under the curve of dEMG signals in the present study, suggesting enhanced force generation by the diaphragm and perhaps increased tidal volume (Graßhoff et al., 2021, 2023). It has to be acknowledged that in most of the studies that reported normoxic hyperventilation after CSH, respiratory factors have been measured in awake animals, but our measurements were done on anaesthetized rats. Thus, VDH after chronic hypoxia appears to be sensitive to anaesthetic agents, a finding that was previously reported (Bonora & Vizek, 2001). In their study, Bonora and Vizek used sodium pentobarbital to induce anaesthesia in rats, and here we used urethane (1.5 g/kg). It has been found that these anaesthetic agents have a depressant effect on respiratory rhythmogenesis (Erhardt et al., 1984; Flecknell, 2016; Irwin et al., 2023; Webster & Karan, 2020). Urethane is known to antagonize glutamatergic NMDA receptors (Hara & Harris, 2002; Massey & Richerson, 2017), and interestingly, it has been shown that CSH treatment enhances glutamate neurotransmission and NMDA receptors in the second‐order chemosensitive neurons of the nucleus of the solitary tract (NTS), which contributes to a time‐dependent increase in ventilation (Pamenter et al., 2014). Since these neurons directly project to preBötC respiratory rhythm‐generating neurons, it is reasonable to assume that utilizing urethane to induce anaesthesia abolishes NMDA‐mediated enhanced ventilation during CSH. However, it remains to be determined why respiratory frequency was lower in CSH rats compared to control normoxic rats. One possibility is that these anaesthetics may have a greater depressant effect on rats treated with CSH, which requires further study to be clarified.

Analyses of dEMG activity in the frequency domain can provide information on motor unit recruitment (Mantilla et al., 2010). Therefore, we aimed to estimate changes in the mean frequency and high‐frequency power to low‐frequency power ratio (H/L ratio) of dEMG signals. We found a significant decrease in these variables following CSH treatment. The frequency content of an EMG signal is influenced by the conduction velocity of recruited muscle fibres (Lindstrom & Magnusson, 1977). A shift in the power spectral density (PSD) of an EMG signal to higher frequencies may reflect the enhanced contribution of motor units with fatigable fast‐twitch muscle fibres (Seven et al., 2013). According to our findings (decreased H/L ratio and mean frequency), chronic exposure to hypoxia probably induced adaptive changes in the diaphragm muscle, such that the contribution of motor units with small and fatigue‐resistant muscle fibres (e.g., type I and IIa fibres) might become greater than that of larger and fatigable fibres (e.g., type IIx and/or IIb fibres) (Geiger et al., 2000; Miyata et al., 1995; Prakash et al., 2000; Sieck et al., 1989). These findings support previous studies that reported improved fatigue tolerance (suggestive of increased aerobic capacity) in rodent diaphragm after sustained hypoxia (El‐Khoury et al., 2003; Gamboa & Andrade, 2012; McMorrow et al., 2011; Shiota et al., 2004). Moreover, the mean frequency of an EMG signal is inversely proportional to muscle force and is also sensitive to the synchronization of motor units, such that during increased muscle force or enhanced coherence between motor units, the mean frequency decreases (McManus et al., 2016; Thongpanja et al., 2013). This is in line with our findings regarding increased total power and estimated muscle force associated with decreased mean frequency of dEMG signals.

Changes in the regularity of inspiratory bursts from preBötC to respiratory motoneurons have been previously reported following intermittent (episodic) hypoxic stimulus (Garcia et al., 2016; Zanella et al., 2014). Thus, we decided to evaluate the regularity of dEMG bursts following CSH. Although CSH led to increased irregularity in both frequency (period) and amplitude, these changes were not statistically significant. The respiratory rhythm irregularity following chronic intermittent hypoxia might be related to brainstem oxidative stress, since antioxidant supplementation mitigates chronic intermittent hypoxia‐mediated irregularities in rhythmogenesis (Garcia et al., 2016). Notably, in our previous work on rats, we reported increased oxidative stress in the brainstem following CSH (Khalilpour et al., 2024). Thus, increased irregularities, although insignificant, in dEMG bursts may be attributable to increased brainstem oxidative stress following CSH.

### 4.1 Conclusions

In summary, chronic sustained hypoxia for 10 consecutive days led to significant changes in the pattern of dEMG bursts and the characteristics of dEMG signals. The slope of the rising phase of RMS‐enveloped dEMG bursts was much steeper in CSH rats compared with normoxic control rats, which was responsible for the altered dEMG burst shape. Furthermore, burst frequency, burst amplitude and inter‐burst duration had significantly changed following CSH. Analyses of dEMG signals in the frequency domain showed that CSH significantly decreased the mean frequency and high‐frequency power to low‐frequency power ratio (H/L ratio) of dEMG signals, which might result from altered recruitment pattern of diaphragm motor units by the respiratory network. Notably, we did not observe significant changes in the regularity of frequency and amplitude of dEMG signals after CSH treatment.

## AUTHOR CONTRIBUTIONS

Jamal Khalilpour and Parviz Shahabi conceived the study and designed the experiments. Jamal Khalilpour wrote the manuscript. Jamal Khalilpour and Parviz Shahabi performed the experiments. Jamal Khalilpour, Parviz Shahabi and Mohammad Reza Alipour interpreted data and contributed to the discussion. All authors reviewed and concurred with the final manuscript. Parviz Shahabi takes responsibility for the integrity of the data and the accuracy of the data analysis. All authors have read and approved the final version of this manuscript and agree to be accountable for all aspects of the work in ensuring that questions related to the accuracy or integrity of any part of the work are appropriately investigated and resolved. All persons designated as authors qualify for authorship, and all those who qualify for authorship are listed.

## CONFLICT OF INTEREST

None declared.

## Data Availability

The datasets used and analysed during the current study are available from the corresponding author upon reasonable request.
